# Does digital cognitive behavioral therapy improve the insomnia and depression of workers to healthy levels? An open trial

**DOI:** 10.1186/s13030-025-00334-y

**Published:** 2025-07-21

**Authors:** Isa Okajima, Miho Suzuki, Noriko Tanizawa, Ikuo Kajiyama, Reiko Ichikawa, Jou Akitomi

**Affiliations:** 1 Behavioral Sleep Medicine and Sciences Laboratory, Department of Psychological Counseling, Faculty of Humanities, Tokyo Kasei University, Tokyo, 173-8602 Japan; 2 NEC Solution Innovators Ltd, Tokyo, 136-8627 Japan; 3 MEDIVA Inc, Tokyo, 154-0023 Japan

**Keywords:** Cognitive behavioral therapy, Insomnia, Depression, Digital, Social disability, Presenteeism

## Abstract

**Background:**

Cognitive behavioral therapy for insomnia (CBT-I) has a high potential for improving insomnia and depressive symptoms; however, it is unclear whether the improvement of symptoms reaches the level of healthy people without these symptoms. We aimed to examine whether digital CBT-I can improve the insomnia and depression symptoms of workers to healthy levels.

**Methods:**

The study included 752 workers who were divided into four groups by the use of the cutoff scores of insomnia and depression scales: insomnia alone, depression alone, combined insomnia with depression (COMB), and healthy. All groups were administered digital CBT-I for 2 weeks, and changes were compared post-treatment and at 1- and 3-month follow-ups.

**Results:**

A significant decrease in insomnia symptoms from post-treatment to the 3-month follow-up was found in the insomnia alone (Hedges’ g: 1.07–1.52) and COMB groups (g: 1.17–1.41). The COMB group also showed a significant decrease in depressive symptoms (g: 0.38–0.70). Moreover, there were significant differences in insomnia symptoms between both of the insomnia groups and the healthy group and in depressive symptoms between the COMB group and the healthy group, post-treatment and at the 1- and 3-month follow-ups.

**Conclusions:**

Digital CBT-I effectively reduced insomnia and depressive symptoms but did not achieve the levels of healthy people within 3 months.

Trial registration.

UMIN, UMIN000050353. Registered 15 February 2023—Retrospectively registered, umin.ac.jp/ctr.

**Supplementary Information:**

The online version contains supplementary material available at 10.1186/s13030-025-00334-y.

## Background

It is reported that one to two-fifths of the general population have experienced insomnia symptoms [1–3] and that one-fifth of them develop a chronic insomnia disorder [3]. Chronic insomnia can last a lifetime and is linked to the onset or recurrence of depression [4–6]. Nearly 80% of patients with depression have initial, middle, and terminal insomnia as nocturnal symptoms [7].

Since the coronavirus disease (COVID-19) outbreak, insomnia and depression have become serious health issues. In an international collaborative study [8], approximately 40% of a community sample complained of insomnia. Insomnia symptoms during the outbreak were related to post-traumatic stress and psychological distress [9]. Additionally, COVID-19 infection-related stress and worry have been linked to insomnia, depression, and anxiety [10]. The chance of developing post-onset insomnia problems is also higher in people with COVID-19 than in people with other viruses, such as the flu [11, 12].

Cognitive behavioral therapy for insomnia (CBT-I) is a highly successful first-line intervention for persistent insomnia disorder [13–15], with CBT-I in both face-to-face and digital formats having been shown to be effective [16, 17]. Two randomized controlled trials revealed that digital CBT-I improved nighttime (insomnia severity, pre-sleep arousal, dysfunctional beliefs about sleep, and sleep reactivity) and daytime (quality of life, well-being, and work productivity) aspects of insomnia [16, 17]. In a systematic review and meta-analysis, digital CBT-I significantly reduced insomnia severity, sleep onset latency (SOL), wake after sleep onset (WASO), and sleep efficiency (SE) for both clinical (patients) and industrial (non-patients) settings [18, 19]. Other studies of these settings have shown that the improvement in the severity of insomnia when using a digital format was inferior to that when using a face-to-face format [18, 19]. In particular, digital CBT-I has been reported to sufficiently improve insomnia severity, particularly in workers, younger adults, and individuals with mild insomnia [20]. A stepped care model of CBT-I reported the effectiveness of self-help therapeutics including fully-automated digital CBT-I in first level treatment, and these programs were proposed for use in the workplace as a preventive for insomnia [21]. The use of digital CBT-I formats has played a key role in treatment, especially since the COVID-19 outbreak.

In addition, CBT-I improves the symptoms of insomnia in patients with major depressive disorder [22, 23]. Two randomized controlled trials on CBT-I that included patients with comorbid major depression and insomnia disorder revealed that CBT-I improved insomnia and depressive symptoms post-treatment and at a 1-month FU [22, 23]. CBT-I considerably lessened insomnia and depressive symptoms in a systematic review and meta-analysis when compared to controls, and the improvements were sustained during the FU period [24, 25]. Thus, CBT-I has a high potential for improving insomnia and psychiatric symptoms.

Previous studies on CBT-I in patients with comorbid depression and insomnia have not clarified the following aspects: (1) whether CBT-I directly or indirectly leads to the improvement of depressive symptoms and (2) whether the improvement of the symptoms of insomnia and depression reach the levels of healthy of people without these symptoms.

On the first issue, face-to-face CBT-I and control therapy can partially improve depressive symptoms [26, 27]. However, it is unclear whether CBT-I techniques or expert support directly affect insomnia, depressive symptoms, or both in individuals with comorbid depression and insomnia. A study using a full-automatedly digital CBT-I is appropriate for examining the unique effects of CBT-I without the influence of expert support. On the second issue, individuals with comorbid depression and insomnia were divided into intervention and control groups in numerous studies to examine the effects of CBT-I, and comparisons were made between patient groups [22, 23, 26, 27]. To examine the level of improvement in symptoms and daytime function after CBT-I, comparisons with a healthy group are essential.

In this study, we aimed to examine how digital CBT-I affects insomnia and depressive symptoms, social disability, and work productivity among workers with insomnia alone, depression alone, or comorbid insomnia and depression in comparison with a healthy group.

## Methods

### Participants

From June 1, 2020, to September 9, 2020, we recruited volunteers via websites and leaflet distribution at NEC group-affiliated companies in Japan. Daytime workers who took part in the study completed an online questionnaire and an informed consent form. The inclusion criteria were as follows: (1) workers aged 20 years or older and (2) workers who were interested in improving their sleep and were not aware of our study project. Shift-workers were excluded from this study.

A total of 835 full-time or part-time workers (mean [standard deviation: SD] age, 45.17 [11.01] years; 500 men, 335 women) responded to our recruitment ads; however, the data of 83 (9.9%) were excluded because they had not provided informed consent. A total of 752 workers (mean [SD] age, 44.34 [10.96] years; 454 men, 298 women) were eligible for the study, including 253 participants assigned to the insomnia alone group (monthly overtime hours [SD]; 19.3 [20.5]), 39 participants to the depression alone group (12.2 [13.3]), 296 participants to the combined insomnia with depression (COMB) group (18.3 [21.3]), and 164 participants to the healthy group (15.6 [18.2]).

### Assessment measures

Participants were assessed using an online form given pre- and post-intervention and at 1- and 3-month FUs. The primary outcomes were a total Athens Insomnia Scale (AIS) score [28–30] indicating insomnia severity, and a total Kessler Psychological Distress Scale (K6) score indicating depression [31, 32]. The AIS and K6 comprise eight and six items, respectively, and the total score was calculated for each. The clinical cutoff score of AIS was 5.5 points [28, 30]. When the K6 score was ≥ 5 points, the participant is judged as having a level from psychological distress to depression [32]. Higher scores on both scales indicate worse symptoms.

Secondary outcomes were social disability measured using the Sheehan Disability Scale (SDISS) [33], daytime sleepiness measured using the Epworth Sleepiness Scale (ESS) [34], and work productivity affected by presenteeism measured using the World Health Organization (WHO) Health and Work Performance Questionnaire (HPQ) [35]. The SDISS comprises three domains that assess daytime disabilities in work performance, social life, and family life. The ESS consists of eight items about the propensity to doze off while sedentary. The HPQ score was calculated by multiplying the raw score by 10 (range, 0–100). Higher scores on both SDISS and ESS indicated worse daytime function, whereas lower scores on the HPQ indicated worse work productivity.

### Study design

**Fig. 1:**
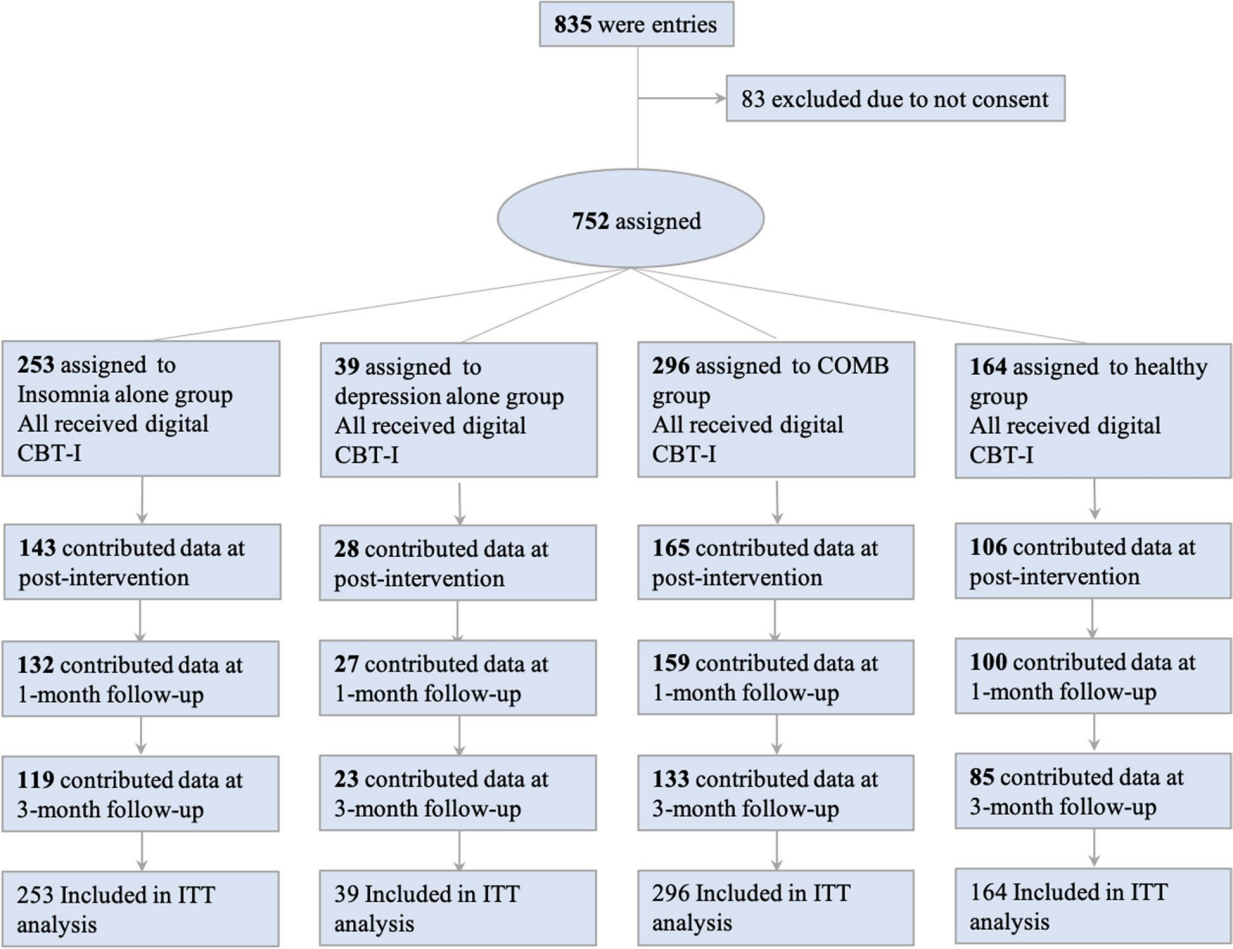
Study flow chart

A prospective parallel-group design was adopted in this open trial (Fig. 1). Based on the AIS and K6 cutoff scores, participants were divided into four groups as follows: Healthy (AIS < 6 and K6 < 5), insomnia alone (AIS ≥ 6 and K6 < 5), depression alone (AIS < 6 and K6 ≥ 5), and COMB (AIS ≥ 6 and K6 ≥ 5). Each participant was provided a tailored digital CBT-I validated by Okajima, et al. [17].

Participants were provided with information about the background and purpose of the study, voluntary participation, the type of research intervention, the duration, risks, benefits, confidentiality, sharing of the findings, the right to refuse or withdraw from the study, alternative ways to participate, and who to contact regarding informed consent. They were asked to sign an agreement form if they agreed with all the content.

Written informed consent was obtained from each participant, and the study was carried out in accordance with the Declaration of Helsinki and was approved by the Ethics Committee of NEC Life Science (protocol code LS2018-004; Trial registration: umin.ac.jp/ctr Identifier: UMIN000050353).

### Sample size

A power analysis was done based on the findings of a previous study that used a tailored digital CBT-I application [17]. At the 1-month FU, effect sizes (Hedges’ g) for the severity of insomnia were 0.85 [17]. We determined that 43 data sets would be needed for each group (total *N* = 172) to show a significant difference at = 0.001 (two-sided) with a power of 0.9. Because the previously reported dropout rate for the application was primarily during the 3-month FU period [17], we allowed for a 50% dropout rate, which necessitated the recruitment of 86 participants per group (for a total of 344 participants). In addition, because the ratio of depression with insomnia to depression without insomnia is approximately 8:2 [7], it was possible that an insufficient number of individuals would be assigned to the depression alone group. Therefore, more than 344 participants were recruited.

### Interventions

Each participant downloaded the application for the tailored digital CBT-I on their Android or iOS smartphone [17]. The application is a two-week program that includes 26 challenge tasks that were developed based on CBT-I techniques for stimulus control, sleep restriction, relaxation, and sleep hygiene. Participants chose 1 to 3 of the tasks that had been suggested (i.e., tailored to oneself) to them based on the baseline assessments, then they focused on these tasks for the 2 weeks of the program (for program details [17]). All participants were assessed for sleep habits (bed/wake time, physical activity, and exposure to bright light) at pre- and post-interventions and at 1- and 3-month FUs.

### Statistical analysis

Chi-square test (group × sex) and an analysis of variance for age between groups were used to examine whether sex and age were potential confounding variables. The results showed a significant difference in age (F_3,748_ = 5.29, *p* < 0.01, η^2^ = 0.02). Therefore, we tested the goodness of fit of the model with vs. without age, and because the AIC of the model without age was better (9193.3 vs. 9191.3), we conducted a linear mixed model that excluded sex and age. A majority of the analyses used an intent-to-treat paradigm. A linear mixed model without missing value imputation was used to analyze pre- and post-intervention and 1- and 3-month FU data in all groups to examine the impact of tailored digital CBT-I on insomnia, depressive symptoms, daytime disabilities, sleepiness, and productivity. We applied the Bonferroni–Holm adjustment for p-values when the main or interaction effects in all analyses were shown, and then we performed post hoc analyses. To reduce the possibility of finding significant p-values as a result of multiple post hoc analyses, we introduced the Bonferroni–Holm correction for p-values.

By adjusting the biases for Hedges’ g, we also calculated the effect sizes of the scales within and between the groups. A small effect size was indicated by an absolute g value of at least 0.2, a moderate effect size was about 0.5, and a large effect size was at least 0.8 [36]. Comparisons with pre- vs. post-intervention, pre-intervention vs. 1-month FU, and pre-intervention vs. 3-month FU were used to calculate the effect sizes of all scales within the groups. The effect sizes of the insomnia alone and COMB groups were compared with the healthy group, post-intervention and at the 1- and 3-month FUs.

R statistical software, version 3.4.4 (R Project for Statistical Computing, Vienna, Austria) was used, and descriptive statistics were by lmerTest [37], lsmeans [37], and compute.es [38].

## Results

An analysis of variance test was done to compare a participant-only model with a model that included a participant × group interaction for the random-effect linear mixed model for all scales. This was necessary to account for the individual differences of each participant while controlling for the group because there are individual differences, even among participants in the same group. The result of the χ^2^ test was significant (ps < 0.001) and the AICs with the interaction model were better than those without the interaction model for all scales (e.g., the AIS and K6 as primary outcomes: AIC = 9204.95 vs. 9552.18 and AIC = 9546.96 vs. 9976.67). Therefore, we adopted a more complicated model (interaction model). For the fixed-effect linear mixed model for all scales except the HPQ, the interaction effects were significant: AIS (F_9, 1345_ = 19.89, *p* < 0.001), K6 (F_9, 1330_ = 8.49, *p* < 0.001), SDISS-work performance (F_9, 1277_ = 4.54, *p* < 0.001), SDISS-social life (F_9, 1278_ = 3.54, *p* < 0.001), SDISS-family life (F_9, 1254_ = 2.07 *p* = 0.03), and ESS (F_9, 1294_ = 2.32, *p* = 0.01). For the HPQ, both main effects were significant (group: F_3, 697_ = 49.16, *p* < 0.001, time: F_3, 1336_ = 44.69, *p* < 0.001). Based on these results, we examined the results of all of the scales in detail. Descriptive statistics of all scales for each group pre- and post-treatment and at the 1-month and 3-month FUs are shown in Table S1.

### Effect of digital CBT-I on the main outcomes

**Fig. 2:**
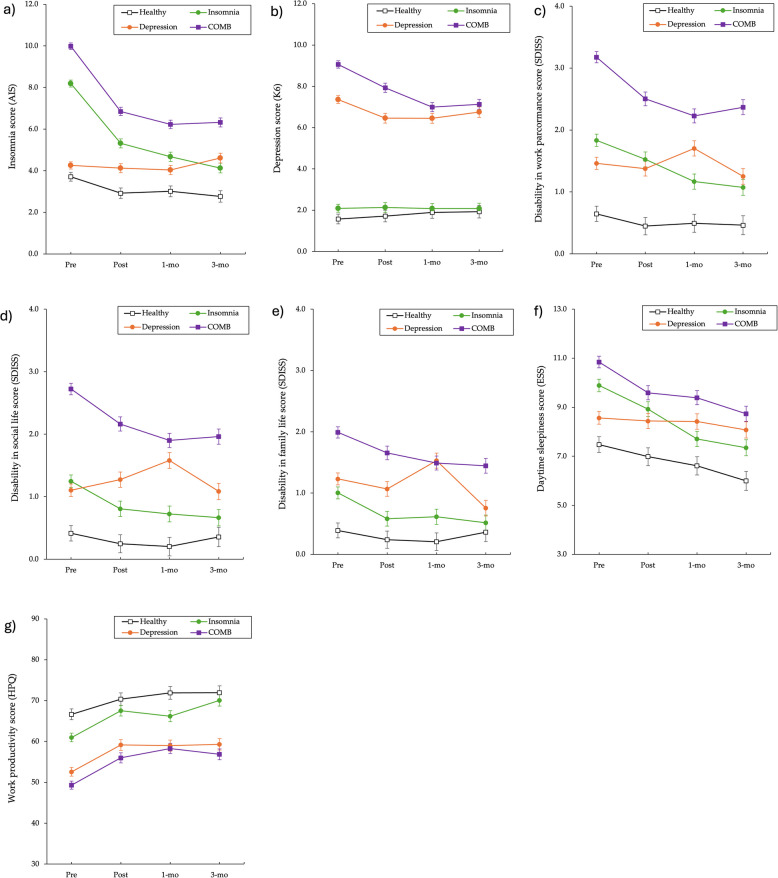
Changes in the scores for each scale: (**a**) insomnia severity, (**b**) depressive symptom, (**c**) disability in work performance, (**d**) disability in social life, (**e**) disability in family life, (**f**) daytime sleepiness, and (**g**) work productivity. Error bars indicate standard error

**Fig. 3:**
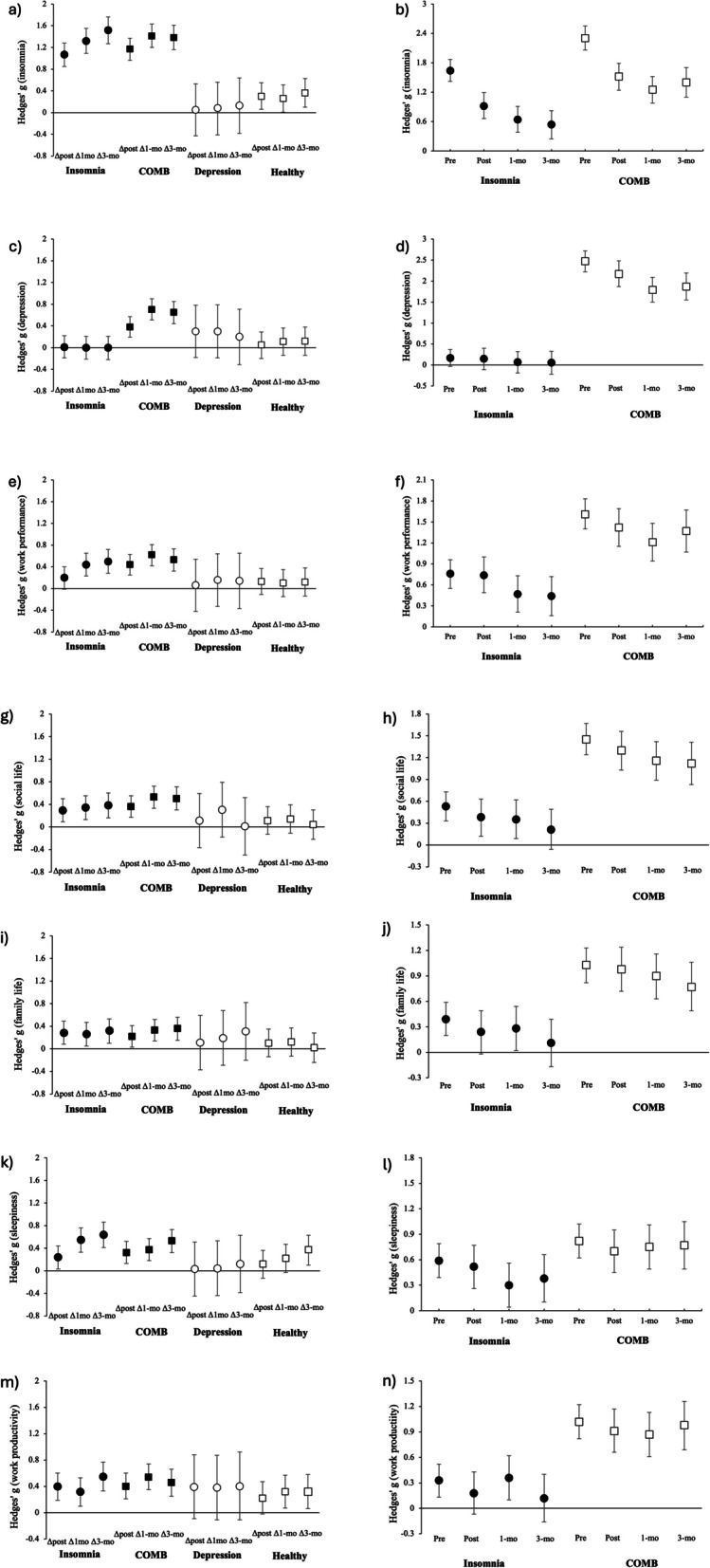
Effect sizes of each scale within (left columns [a, c, e, g, i, k, and m]; vs. pre-treatment) and between (right columns [b, d, f, h, j, l, and n]; vs. healthy group) the groups. **a** Insomnia severity within the groups, (**b**) insomnia severity between the groups, (**c**) depressive symptom within the groups, (**d**) depressive symptom between the groups, (**e**) disability in work performance within the groups, (**f**) disability in work performance between the groups, (**g**) disability in social life within the groups, (**h**) disability in social life between the groups, (**i**) disability in family life within the groups, (**j**) disability in family life between the groups, (k) daytime sleepiness within the groups, (**l**) daytime sleepiness between the groups, (**m**) work productivity within the groups, and (**n**) work productivity between the groups. In the left columns, positive values indicate improvement of symptom and daytime disabilities. In the right columns, the closer to 0, the less difference from the healthy group. Error bars indicate 95% CIs of Hedges’ g

For the severity of insomnia, the AIS scores significantly reduced from pre-treatment to post-treatment and from the 1-month to the 3-month FU (ps < 0.001; Fig. 2a). Effect sizes were large within the insomnia alone (g [95% CI] = 1.07 [0.86, 1.29], 1.32 [1.09, 1.55], and 1.52 [1.28, 1.77], respectively) and COMB groups (g = 1.17 [0.97, 1.38], 1.41 [1.19, 1.62], and 1.38 [1.15, 1.60], respectively; Fig. 3a). However, the AIS scores of both groups remained higher than those of the healthy group post-treatment and at the 1- and 3-month FUs (ps < 0.001; Fig. 2a). In the insomnia alone group, the effect size changed from large post-treatment (g = 0.92 [0.66, 1.19]) to moderate (g = 0.54 [0.25, 0.82]) at the 3-month FU, as compared to the healthy group (Fig. 3b). In the COMB group, the effect size remained large from post-treatment (g = 1.52 [1.24, 1.79]) to the 3-month FU (g = 1.40 [1.10, 1.70]; Fig. 3b). The AIS scores within the depression alone group and between the depression and healthy groups were not significantly different.

For depressive symptoms, the K6 scores were significantly reduced from pre- to post-treatment and at the 1- and 3-month FUs (*p* < 0.001; Fig. 2b), and the effect sizes were small to large within the COMB group (g = 0.38 [0.19, 0.57], 0.70 [0.50, 0.89], and 0.65 [0.45, 0.86], respectively; Fig. 3c). However, the K6 score remained higher in the COMB group than in the healthy group post-treatment and at the 1- and 3-month FUs (ps < 0.001; Fig. 2b). Additionally, the effect size of the COMB group remained large from post-treatment (g = 2.17 [1.87, 2.48]) to the 3-month FU (g = 1.87 [1.55, 2.19]; Fig. 3d). The K6 scores within the insomnia and depression alone groups were not significantly different.

### Effect of digital CBT-I on secondary outcomes

For disability in work performance, the SDISS-work performance scores were significantly reduced from pre-treatment to post-treatment and at the 1- and 3-month FUs (ps < 0.01; Fig. 2c), and effect sizes were small to moderate within insomnia alone (g [95% CI] = 0.20 [0.00, 0.41], 0.44 [0.23, 0.65], and 0.50 [0.28, 0.72], respectively) and COMB groups (g = 0.44 [0.25, 0.63], 0.62 [0.43, 0.82], and 0.53 [0.33, 0.74], respectively; Fig. 3e). However, the SDISS-work performance scores of both groups remained higher than those of the healthy group post-treatment and at the 1- and 3-month FUs (ps < 0.01; Fig. 2c). In the insomnia alone group, effect size changed from moderate post-treatment (g = 0.74 [0.49, 1.00]) to small (g = 0.44 [0.16, 0.72]) at the 3-month FU, compared with the healthy group (Fig. 3f). In the COMB group, the effect sizes remained large from post-treatment (g = 1.42 [1.15, 1.69]) to the 3-month FU (g = 1.37 [1.07, 1.67]; Fig. 3f). The SDISS-work performance scores were not significantly different in the depression alone and healthy groups.

For disability in social life, the SDISS-social life score was significantly reduced from pre-treatment to post-treatment and at the 1- and 3-month FUs (ps < 0.001; Fig. 2d). Effect sizes were small to moderate within the insomnia alone (g [95% CI] = 0.29 [0.08, 0.49], 0.34 [0.13, 0.55], and 0.38 [0.16, 0.60], respectively) and COMB groups (g = 0.36 [0.17, 0.55], 0.53 [0.34, 0.73], and 0.50 [0.29, 0.70], respectively; Fig. 3g). However, the SDISS-social life score remained higher in the insomnia alone group post-treatment and at the 1-month FU (ps < 0.05) and in the COMB group post-treatment and at the 1- and 3-month FUs (ps < 0.001), compared with the healthy group (Fig. 2d). In the insomnia alone group, the effect size remained small from post-treatment (g = 0.38 [0.12, 0.63]) to the 3-month FU (g = 0.21 [0.06, 0.49]) compared with the healthy group (Fig. 3h). In the COMB group, the effect size remained large from post-treatment (g = 1.30 [1.03, 1.56]) to the 3-month FU (g = 1.12 [0.83, 1.41]; Fig. 3h). The SDISS-social life scores of the depression alone and healthy groups were not significantly different.

For disability in family life, the SDISS-family life score was significantly reduced from pre-treatment to post-treatment and at the 1- and 3-month FUs (ps < 0.01; Fig. 2e). Effect sizes were small within the insomnia alone (g [95% CI] = 0.28 [0.07, 0.48], 0.26 [0.05, 0.47], and 0.32 [0.11, 0.54], respectively) and COMB groups (g = 0.22 [0.03, 0.41], 0.33 [0.14, 0.52], and 0.36 [0.16, 0.57], respectively; Fig. 3i). However, the SDISS-family life score remained higher in the COMB group than in the healthy group post-treatment and at the 1- and 3-month FUs (ps < 0.001; Fig. 2e). In the COMB group, the effect size remained large post-treatment (g = 0.98 [0.72, 1.24]) and was moderate at the 3-month FU (g = 0.77 [0.49, 1.06]; Fig. 3j). The SDISS-family life scores of the depression alone and healthy groups were not significantly different.

For daytime sleepiness, the ESS score was significantly reduced from pre-treatment to post-treatment and at the 1- and 3-month FUs (ps < 0.001; Fig. 2f). Effect sizes were small to moderate within the insomnia alone (g [95% CI] = 0.24 [0.04, 0.45], 0.55 [0.34, 0.77], and 0.64 [0.42, 0.87], respectively) and COMB groups (g = 0.32 [0.12, 0.51], 0.37 [0.17, 0.56], and 0.53 [0.33, 0.74], respectively; Fig. 3k). However, the ESS score remained higher in the insomnia group post-treatment and at the 3-month FU (ps < 0.05) and in the COMB group post-treatment and at the 1- and 3-month FUs (ps < 0.001), compared with those of the healthy group (Fig. 2f). In the insomnia alone group, the effect size changed from large post-treatment (g = 0.52 [0.26, 0.77]) to small at the 3-month FU (g = 0.38 [0.10, 0.66]), compared with the healthy group. In the COMB group, the effect size remained moderate from post-treatment (g = 0.70 [0.45, 0.95]) to the 3-month FU (g = 0.77 [0.49, 1.05]; Fig. 3l). The ESS scores of the depression alone and healthy groups were not significantly different.

For work productivity, the effect sizes of HPQ were calculated as reference values because the main effect was significant, although the interaction effect is not (Fig. 2g, Fig. 3m, and Fig. 3n). The effect sizes were small to moderate within the insomnia alone (g [95% CI] = 0.40 [0.19, 0.60], 0.32 [0.10, 0.53], and 0.55 [0.33, 0.77], respectively) and COMB groups (g = 0.40 [0.21, 0.60], 0.54 [0.35, 0.74], and 0.46 [0.25, 0.66], respectively).

## Discussion

In this study, we examined how digital CBT-I affected insomnia and depressive symptoms, social disability, and work productivity among workers with insomnia alone, depression alone, and comorbid insomnia and depression, compared with those in a healthy group. In line with previous reports, our findings revealed that digital CBT-I was significantly related to variables within the groups, and although there were improvements, healthy levels were not achieved.

Digital CBT-I largely improved the insomnia severity of the insomnia alone and COMB groups, and this improvement was maintained for 3 months. These results are consistent with those of previous studies [17, 39]. In the COMB group, digital CBT-I was also effective for depressive symptoms post-treatment, and the effect was maintained for 3 months, although the effect sizes were small to large. These results are in line with those of previous studies on depression comorbid with insomnia [22, 23]. In contrast, the insomnia severity and depressive symptoms of the depression alone group did not improve. Thus, CBT-I, including either the digital or face-to-face format, was shown to improve insomnia severity directly and that it may subsequently reduce depressive symptoms. Mediation analyses have revealed that CBT-I decreases depressive symptoms by alleviating insomnia symptoms [26, 39, 40].

Insomnia is related to the onset, maintenance, and relapse of depression [4, 41], and digital CBT-I for people with insomnia prevents the onset and recurrence of depressive disorders [42]; thus, CBT-I should be actively provided to patients with insomnia.

Digital CBT-I is highly effective for people with insomnia. In particular, the mean score of AIS reached a level under the cut-off point (≤ 5 points) at one or more months FU in the insomnia group; however, it was still significantly different between the healthy group and the other groups. Considering the overall high social function of the healthy group, the improvement due to digital CBT-I would not be sufficient. Nevertheless, these differences decreased over time, especially regarding insomnia severity. The FU period in this study was set to 3 months; however, a FU period of 1–2 years may have revealed improvement to a healthy level. Otherwise, specific interventions for depression comorbid with insomnia may need to be developed. Depressive insomnia is associated with sleep reactivity, which is defined as the trait-like extent to which exposure to stress interferes with sleep. Meanwhile, anxious insomnia is connected to anxiety sensitivity, which is characterized as fear of unfavorable consequences related to anxiety arousal [43]. An approach to reduce sleep reactivity may be useful for persons with comorbid depression and insomnia because it is a vulnerability factor for the onset of both disorders [44]. Self-help CBT-I has been reported to reduce sleep reactivity, which contributes to improvement in insomnia severity [40]. However, the reason CBT-I reduces sleep reactivity has not yet been properly examined. Further studies are needed to examine specific techniques for reducing sleep reactivity.

In both the insomnia alone and COMB groups, digital CBT-I reduced small-to-moderate daytime dysfunction, including social disabilities and sleepiness, and the reduction was maintained for three months. These results are in line with those of a previous study using the same intervention [17]. Daytime functions were not improved in the depressive-alone group; therefore, our results support the notion that digital CBT-I contributes to daytime function by improving nocturnal symptoms. CBT for the excessive worry of persons with generalized anxiety disorder revealed that the only relevant factor in their improvement in daytime function was a reduction in insomnia, but not worry, cognitive-behavioral avoidance, or intolerance of uncertainty [45]. Thus, an improvement in insomnia leads to a healthier daily life and enhanced work productivity.

The degree of improvement in daytime function did not achieve a healthy level, similar to the symptoms of insomnia and depression. However, these differences decreased over time, especially in the insomnia group. The COMB group may have been more affected during the day because of depressive symptoms; however, a longer FU period may have led to improvement to a healthy level. Furthermore, cognitive components (for example, cognitive restructuring) are more effective in quality-of-life impairment than behavioral components (such as sleep scheduling) [46]. Because the behavioral component was the core program of this study, it will be necessary to include the cognitive component in future studies.

This study has some limitations. First, the study design was not that of a randomized controlled trial. All participants in this study hoped to receive digital CBT-I, and the intervention was provided to them; therefore, it is assumed that selection bias due to treatment motivation was not large. Second, the demographic data are insufficient. All participants worked full-time or part-time; however, treatment efficacy may vary depending on medical history and co-occurrence of other sleep disorders. Finally, we used a cut-off of K6 ≥ 5 for depression to collect the large sample necessary for this study. The cut-off for depression level according to K6 is set at 9 points or higher [32]. In the present study, the mean score of the COMB group was 9 points at baseline, suggesting that the participants were at the level of depression. However, future studies will be necessary to examine if these results are also applicable to more severe depressed patients.

## Conclusions

Digital CBT-I had a positive effect on the nocturnal insomnia symptoms and daytime function of workers with insomnia with/without depression during the COVID-19 outbreak. However, the failure of patients to recover to healthy levels of insomnia and depression could lead to economic losses. Therefore, additional research is required to improve the therapeutic effectiveness of CBT-I.

## Supplementary Information

Supplementary Material  1: Table S1. Descriptive statistics for all scales in each group.

## Data Availability

Data utilized in this study is available upon request from the corresponding author.

## Abbreviations

AIC: Akaike Information Criterion
AIS: Athens Insomnia Scale
CBT-I: Cognitive behavioral therapy for insomnia
COVID-19: Coronavirus disease 2019
ESS: Epworth Sleepiness Scale
FU: Follow-up
HPQ: Health and Work Performance Questionnaire
ITT: Intent-to-treat
K6: Kessler Psychological Distress Scale
RCT: Randomized controlled trial
SDISS: Sheehan Disability Scale
SE: Sleep efficiency
SOL: Sleep onset latency
WASO: Wake after sleep onset
WHO: World Health Organization
