# Supplementary material for: Does digital cognitive behavioral therapy improve the insomnia and depression of workers to healthy levels? An open trial
Source: Biopsychosoc Med. 2025 Jul 21;19:13. doi: 10.1186/s13030-025-00334-y (PMC12278518; doi:10.1186/s13030-025-00334-y)
Supplement: Supplementary file 1 — Supplementary Material 1: Table S1. Descriptive statistics for all scales in each group. [file 13030_2025_334_MOESM1_ESM.docx]

**Table S1.** Descriptive statistics for all scales in each group

|  |  | Healthy | | Depression alone | | Insomnia alone | | COMB^1^ | | Random-effect | Fixed-effect |
| --- | --- | --- | --- | --- | --- | --- | --- | --- | --- | --- | --- |
| Scales |  | M | SE | M | SE | M | SE | M | SE | (interaction) | (interaction) |
| AIS | Pre | 3.71 | 0.21 | 4.26 | 0.44 | 8.19 | 0.17 | 9.99 | 0.16 | *** | *** |
|  | 95% CI | 3.03, 4.40 | | 2.85, 5.66 | | 7.64, 8.74 | | 9.48, 10.50 | |  |  |
|  | Post | 2.92 | 0.25 | 4.12 | 0.50 | 5.32 | 0.22 | 6.85 | 0.20 |  |  |
|  | 95% CI | 2.10, 3.74 | | 2.51, 5.73 | | 4.62, 6.01 | | 6.20, 7.50 | |  |  |
|  | 1-mo | 3.01 | 0.26 | 4.04 | 0.51 | 4.67 | 0.22 | 6.23 | 0.20 |  |  |
|  | 95% CI | 2.17, 3.85 | | 2.40, 5.67 | | 3.95, 5.39 | | 5.57, 6.89 | |  |  |
|  | 3-mo | 2.77 | 0.28 | 4.61 | 0.54 | 4.13 | 0.23 | 6.32 | 0.22 |  |  |
|  | 95% CI | 1.88, 3.66 | | 2.88, 6.34 | | 3.38, 4.88 | | 5.62, 7.03 | |  |  |
| K6 | Pre | 1.57 | 0.24 | 7.36 | 0.49 | 2.09 | 0.19 | 9.06 | 0.18 | *** | *** |
|  | 95% CI | 0.81, 2.34 | | 5.79, 8.93 | | 1.47, 2.70 | | 8.49, 9.63 | |  |  |
|  | Post | 1.71 | 0.28 | 6.46 | 0.55 | 2.13 | 0.24 | 7.93 | 0.22 |  |  |
|  | 95% CI | 0.81, 2.62 | | 4.67, 8.24 | | 1.36, 2.90 | | 7.21, 8.64 | |  |  |
|  | 1-mo | 1.89 | 0.29 | 6.45 | 0.56 | 2.08 | 0.24 | 6.99 | 0.22 |  |  |
|  | 95% CI | 0.97, 2.82 | | 4.64, 8.26 | | 1.29, 2.87 | | 6.27, 7.72 | |  |  |
|  | 3-mo | 1.92 | 0.30 | 6.75 | 0.59 | 2.08 | 0.25 | 7.13 | 0.24 |  |  |
|  | 95% CI | 0.94, 2.90 | | 4.84, 8.66 | | 1.26, 2.90 | | 6.35, 7.90 | |  |  |
| SDISS | Pre | 0.65 | 0.12 | 1.46 | 0.25 | 1.83 | 0.10 | 3.18 | 0.09 |  |  |
| -work performance | 95% CI | 0.25, 1.04 | | 0.65, 2.27 | | 1.52, 2.15 | | 2.88, 3.47 | |  |  |
|  | Post | 0.45 | 0.14 | 1.38 | 0.28 | 1.53 | 0.12 | 2.50 | 0.11 | *** | *** |
|  | 95% CI | -0.01, 0.91 | | 0.47, 2.28 | | 1.14, 1.91 | | 2.14, 2.86 | |  |  |
|  | 1-mo | 0.49 | 0.14 | 1.70 | 0.28 | 1.17 | 0.12 | 2.23 | 0.11 |  |  |
|  | 95% CI | 0.03, 0.96 | | 0.78, 2.62 | | 0.77, 1.56 | | 1.86, 2.59 | |  |  |
|  | 3-mo | 0.46 | 0.15 | 1.25 | 0.30 | 1.07 | 0.13 | 2.37 | 0.12 |  |  |
|  | 95% CI | -0.03, 0.95 | | 0.29, 2.22 | | 0.66, 1.49 | | 1.98, 2.75 | |  |  |
| SDISS | Pre | 0.41 | 0.12 | 1.10 | 0.25 | 1.25 | 0.10 | 2.72 | 0.09 | *** | *** |
| -social life | 95% CI | 0.01, 0.81 | | 0.28, 1.92 | | 0.93, 1.57 | | 2.43, 3.02 | |  |  |
|  | Post | 0.25 | 0.14 | 1.27 | 0.29 | 0.81 | 0.12 | 2.16 | 0.11 |  |  |
|  | 95% CI | -0.22, 0.72 | | 0.35, 2.20 | | 0.41, 1.20 | | 1.79, 2.53 | |  |  |
|  | 1-mo | 0.20 | 0.15 | 1.58 | 0.29 | 0.72 | 0.13 | 1.90 | 0.12 |  |  |
|  | 95% CI | -0.27, 0.68 | | 0.64, 2.52 | | 0.31, 1.13 | | 1.53, 2.27 | |  |  |
|  | 3-mo | 0.36 | 0.16 | 1.08 | 0.31 | 0.66 | 0.13 | 1.96 | 0.12 |  |  |
|  | 95% CI | -0.15, 0.86 | | 0.10, 2.07 | | 0.24, 1.09 | | 1.56, 2.36 | |  |  |
| SDISS | Pre | 0.39 | 0.12 | 1.23 | 0.25 | 1.00 | 0.10 | 1.99 | 0.09 | *** | * |
| -family life | 95% CI | 0.00, 0.78 | | 0.42, 2.04 | | 0.69, 1.32 | | 1.70, 2.28 | |  |  |
|  | Post | 0.24 | 0.14 | 1.07 | 0.28 | 0.58 | 0.12 | 1.65 | 0.11 |  |  |
|  | 95% CI | -0.22, 0.70 | | 0.16, 1.97 | | 0.19, 0.97 | | 1.29, 2.01 | |  |  |
|  | 1-mo | 0.21 | 0.14 | 1.53 | 0.28 | 0.61 | 0.12 | 1.49 | 0.11 |  |  |
|  | 95% CI | -0.26, 0.67 | | 0.61, 2.45 | | 0.21, 1.01 | | 1.13, 1.86 | |  |  |
|  | 3-mo | 0.36 | 0.15 | 0.75 | 0.30 | 0.51 | 0.13 | 1.44 | 0.12 |  |  |
|  | 95% CI | -0.13, 0.85 | | -0.21, 1.72 | | 0.10, 0.93 | | 1.06, 1.83 | |  |  |
| ESS | Pre | 7.48 | 0.32 | 8.57 | 0.66 | 9.89 | 0.26 | 10.84 | 0.24 | *** | * |
|  | 95% CI | 6.44, 8.52 | | 6.42, 10.71 | | 9.05, 10.72 | | 10.07, 11.62 | |  |  |
|  | Post | 6.98 | 0.37 | 8.44 | 0.73 | 8.92 | 0.31 | 9.59 | 0.29 |  |  |
|  | 95% CI | 5.80, 8.17 | | 6.08, 10.79 | | 7.92, 9.92 | | 8.67, 10.52 | |  |  |
|  | 1-mo | 6.61 | 0.37 | 8.42 | 0.74 | 7.71 | 0.32 | 9.39 | 0.29 |  |  |
|  | 95% CI | 5.41, 7.81 | | 6.04, 10.80 | | 6.69, 8.73 | | 8.45, 10.33 | |  |  |
|  | 3-mo | 6.00 | 0.39 | 8.08 | 0.77 | 7.35 | 0.33 | 8.74 | 0.31 |  |  |
|  | 95% CI | 4.74, 7.26 | | 5.59, 10.56 | | 6.30, 8.41 | | 7.75, 9.73 | |  |  |
| HPQ | Pre | 66.65 | 1.32 | 52.56 | 2.71 | 60.99 | 1.06 | 49.32 | 0.98 | *** |  |
|  | 95% CI | 62.37, 70.92 | | 43.80, 61.33 | | 57.55, 64.43 | | 46.14, 52.51 | |  |  |
|  | Post | 70.36 | 1.55 | 59.15 | 3.06 | 67.54 | 1.35 | 55.99 | 1.22 |  |  |
|  | 95% CI | 65.36, 75.36 | | 49.26, 69.04 | | 61.82, 70.55 | | 52.05, 59.94 | |  |  |
|  | 1-mo | 71.88 | 1.58 | 59.00 | 3.10 | 66.19 | 1.35 | 58.27 | 1.24 |  |  |
|  | 95% CI | 66.78, 76.98 | | 48.99, 69.86 | | 61.82, 70.55 | | 54.27, 62.27 | |  |  |
|  | 3-mo | 71.92 | 1.67 | 59.30 | 3.27 | 70.05 | 1.40 | 56.86 | 1.32 |  |  |
|  | 95% CI | 66.53, 77.32 | | 48.74, 69,86 | | 65.52, 74.57 | | 52.60, 61.12 | |  |  |

AIS, Athens Insomnia Scale; ESS, Epworth Sleepiness Scale; HPQ, WHO Health and Work Performance Questionnaire; K6, Kessler Psychological Distress Scale; M, least-squares mean; SDISS, Sheehan Disability Scale; SE, standard error.

^1^ COMB indicates a combination of insomnia and depression.

* *p* < 0.05, *** *p* < 0.001
